# Thinger.io: An Open Source Platform for Deploying Data Fusion Applications in IoT Environments

**DOI:** 10.3390/s19051044

**Published:** 2019-03-01

**Authors:** Alvaro Luis Bustamante, Miguel A. Patricio, José M. Molina

**Affiliations:** Applied Artificial Intelligence Group, Universidad Carlos III de Madrid, Avda. Gregorio Peces-Barba y Martínez, 22, Colmenarejo, 28270 Madrid, Spain; alvarolb@Thinger.io (A.L.B.); molina@ia.uc3m.es (J.M.M.)

**Keywords:** IoT middleware, scalabillity, data fusion applications

## Abstract

In the last two decades, data and information fusion has experienced significant development due mainly to advances in sensor technology. The sensors provide a continuous flow of data about the environment in which they are deployed, which is received and processed to build a dynamic estimation of the situation. With current technology, it is relatively simple to deploy a set of sensors in a specific geographic area, in order to have highly sensorized spaces. However, to be able to fusion and process the information coming from the data sources of a highly sensorized space, it is necessary to solve certain problems inherent to this type of technology. The challenge is analogous to what we can find in the field of the Internet of Things (IoT). IoT technology is characterized by providing the infrastructure capacity to capture, store, and process a huge amount of heterogeneous sensor data (in most cases, from different manufacturers), in the same way that it occurs in data fusion applications. This work is not simple, mainly due to the fact that there is no standardization of the technologies involved (especially within the communication protocols used by the connectable sensors). The solutions that we can find today are proprietary solutions that imply an important dependence and a high cost. The aim of this paper is to present a new open source platform with capabilities for the collection, management and analysis of a huge amount of heterogeneous sensor data. In addition, this platform allows the use of hardware-agnostic in a highly scalable and cost-effective manner. This platform is called Thinger.io. One of the main characteristics of Thinger.io is the ability to model sensorized environments through a high level language that allows a simple and easy implementation of data fusion applications, as we will show in this paper.

## 1. Introduction

Currently, data and information fusion is a very broad field of research in many domains. Modern fusion systems are oriented to the integration of multisensor data, information available in databases, experience and expert knowledge, contextual information, mission status, etc., in order to provide real-time estimates with a high value added about the situations of interest. The current trends in modern Data Fusion (DF) applications are related with the capacity to integrate all types of information or knowledge: observational data, knowledge models (a priori or inductively learned), and contextual information. Each type of information has a different nature and potential support to the output of the fusion process:

- **Observational Data:** Observational data are the original data about the dynamic scenario, as collected from heterogeneous sensors (each one with some observational capability). These data are related to observable entities in the real world that are considered of interest.
- **Contextual Information:** The “context” and information about Contextual Information [1] is defined as “the set of circumstances surrounding a task that are potentially of relevance to its completion.” This information should be considered in the fusion process for this relevance. When the fusion system has to estimate or infer tasks, this process implies the development of a set of best-possible estimation. These estimations should take into account this lateral knowledge [2]. Context could be seen as the framework, i.e., not the specific entity, event, or behavior of main interest but context is the information which is relevant to the formation of a best estimate of these items.
- **Learned Knowledge:** There are situations where a priori knowledge for DF process development cannot be stablished. In this case, one possibility is to try and extract the knowledge through online machine learning processes operating on observational data and context information. These are procedural and algorithmic methods for discovering relationships among targets and behaviors of entities of interest [3]. There is a trade-off involved in trying to develop fully-automated algorithmic DF processes for complex problems where the insertion of human intelligence at some point in the process may be a much more judicious choice.

DF applications can benefit from new Internet of Things (IoT) technologies with the ability to acquire and process a huge amount of heterogeneous sensor data [4].

The possibilities offered by IoT systems are numerous, and can be applied to several fields, from Smart Homes [5], Smart Cities [6], Connected Car [7], Industry 4.0 [8], Smart Farming [9], etc. In general, the IoT can be applied to any process or environment which requires monitoring or remote actuation, generally over the Internet. It can be used for monitoring fuel consumption, control cultivation processes, supervise storage conditions, control pollution, irrigation systems, etc. There are also many recent developments related to monitoring chronic patients or elderly people (associated to the HealtCare term), agriculture (Smart Farming), power generation systems, etc.

Despite recent advances in the IoT infrastructure, capturing, storing and processing a large amount of data from heterogeneous sensors remains, to this day, a challenge. The exponential increase in the number and variety of sensors hinders interoperability for the development of data fusion applications. Modern data fusion applications require the integration of three technologies: Cloud, Big Data and IoT technologies. IoT and Big Data technologies are closely related. The arrival of this large number of connected devices causes the generation of many data that must be analyzed through Big Data technology. Smart objects offer millions of data due to the increase in the number of devices connected to each other. All these data can be managed by massive data analysis techniques. IoT technology, thanks to its connectivity, provides infinite possibilities for Big Data. This interconnectivity and the flow of processes that take place between devices, and their own use, generates a lot of data and those events are produced in real time.

The IoT and Cloud technology have had a dizzying evolution in recent years. Cloud technology allows services to be offered through Internet connectivity. Cloud technology provides the capacity of an on-demand pool of secure, easily accessible, computing resources with good maintenance. In the field of IoT technology, the previous computing capabilities are ideal for an environment characterized by devices with low computing and storage capacities.

Therefore, the aim of this paper is to show the characteristics of a new open source platform called Thinger.io that allows the integration of Cloud, Big Data and IoT technologies for the deployment of data fusion applications capable of collecting, managing and analyzing large volumes of data coming from sensors in a highly scalable and cost-effective manner. Thinger.io incorporates a simple modeling language that allows the deployment of data fusion applications where the integration of these three technologies is necessary. It is even easy to store the information collected in an ecosystem in the cloud, and then model the behavior of sensors, in order to be used in future simulations. In addition to the modeling capabilities that we will see in more detail throughout the paper, Thinger.io is a research effort to substantially and technologically overcome the existing IoT platforms. As will be shown in the following sections, Thinger.io is a technological advance regarding transmission efficiency, real-time bidirectional communication, interoperability and simplicity in the deployment of data fusion applications.

This paper is organized with an opening section that presents current challenges for the deployment of modern data fusion applications. One of them is the ability to model services and applications with these three technologies. The next section describes the architecture of the Thinger.io platform and its capabilities, in order to show part of the modeling language used by the platform. A proof of concept shows the simplicity of modeling a service on Thinger.io based on a weather station. Finally, the paper ends with the most important conclusions and future research directions.

## 2. Challenges for the Deployment of Modern Data Fusion Applications

In modern data fusion applications, the integration of the worlds of Cloud, Big Data and IoT is called for due, mainly, to their ability to complement each other. IoT technology is characterized by being composed of small devices deployed in an extensive geographical area, which have limitations in terms of storage, communication and/or computing capabilities. On the other hand, Cloud technology is characterized by having an almost unlimited capacity of these resources, so it seems logical that both can be complemented. In this way, in recent years, terms are emerging that link these two technologies such as CloudIoT [10], SaaS (Sensing as a Service) [11], SAaaS (Sensing and Actuation as a Service) [12], SEaaS (Sensor Event as a Service) [13] or SenaaS (Sensor as a Service) [14].

Big Data technology benefits from the two previous technologies, in the sense that IoT is the source of the data analyzed and Cloud provides the resources to perform the analysis, all with the aim of analyzing large volumes of data.

This ecosystem of technologies presents a set of challenges that we will analyze below:

- **Monitoring a complex environment:** The first one is related to the ability to monitor a highly dense environment. Thanks to new advances in the field of sensors, there are currently several sensors that allow to capture different parameters such as air quality, acceleration, location of people, humidity, ultraviolet radiation, etc. [15,16]. It is necessary to select the type of sensors to be integrated in our data fusion applications. In some cases, for example, as in wearables [17], integration into devices with limited dimensions will be necessary. These physical limitations will involve the work of small devices with low computing resources and autonomy. As we will see later, Thinger.io has been designed to improve the energy efficiency of the devices on which it works.
- **Connectivity:** At present, we find a multitude of devices that can be connected to the Internet through different technologies: wireless networks (WiFi, Bluetooth 4.0, 6LoWPAN), mobile networks (4G LTE), wired networks (Ethernet, fiber optic), etc. [18]. This variety of interfaces allows us to use a variety of sensors in our data fusion applications, all from different manufacturers, which is a technological challenge in the technologies that support [19]. This involves working with optimized network protocols to work with sensors with limited resources, dealing with the scalability of computing architectures to support the concurrent connection of millions of devices and the availability of permanent access required by such devices. In addition, it is necessary to think of standard forms of communication between devices, self-discovery, etc. In this way, devices from different manufacturers can interact with each other to generate the expected intelligent behavior. Thinger.io has been designed to allow connectivity to a large number of devices, and its connectivity capacity is now growing due to its open nature.
- **Modeling:** One of the main challenges in the integration of these three technologies is the ability to be able to obtain information from the sensors. The IoT environment is characterized by having to gather information from highly heterogeneous devices, technologies and protocols. For an engineer to be able to model a solution in which these three technologies are involved, he/she needs to have knowledge of the different protocols involved, such as message queue telemetry transport (MQTT) or data distribution service (DDS) [20]. Also, we will have to know the platforms that allow the storage and processing of the data coming from the sensors [21]. In this sense, this paper will demonstrate the simplicity of the development and deployment of data fusion applications through Thinger.io.
- **Autonomy:** Autonomy is an important challenge since many data fusion applications require the deployment of distributed sensors and actuators in a geographic area, which are powered by batteries. Therefore, it is necessary to carry out optimizations at different levels of the IoT architecture to extend the lifetime of an implementation [22]. It is possible to optimize in different areas such as protocols to save bandwidth, detection periods, energy saving mode of devices and sensors, etc. [23]. As we will see later, Thinger.io has been designed for the use of a highly optimized protocol that reduces the delay when sending information to the cloud, saves bandwidth, reduces memory space and saves the device’s battery.
- **Security:** Devices deployed in our environment can monitor personal information, or even interact with our resources and nearby elements, like those present in homes or the industry [24]. As such, the security in the IoT ecosystem is a real concern, to avoid leaking sensitive data, or granting access to non-authorized actors to actuate over our environment, like our home, or car. Thus, it is required to have secure clouds, secure connections, anonymity in the information stored in the cloud, etc. [25]. Thinger.io provides a layer of security to avoid granting access to unauthorized actors.

There are several platforms trying to satisfy the increasing demand of connected devices, as well as trying to solve some of the aforementioned challenges. There are many global manufacturers that are developing their own platforms for building IoT ecosystems—from Facebook with its Parse platform, Samsung with the ARTIK technology, ARM with ARM mbed, to other emerging platforms such as Golgi, Temboo, Xively, etc. All of these platforms are proprietary and closed, meaning that they are confined to a cloud deployment managed by a third party. They cannot be deployed inside a private business, industry, or being used without approved devices and sensors for the platform. So, the alternative is to rely on open source initiatives such as Thinger.io, Kaa, ThingSpeak, Particle, or OpenRemote. These platforms can be easily used and adapted for academic, industry, and scientific research. In the following section, after a description of the Thinger.io platform, the research efforts that it incorporates will be discussed. These research efforts focus on the transmission efficiency, real-time bidirectional communication, interoperability and the ease of modeling data fusion applications in ecosystems where the integration of IoT, Cloud and Big Data technologies is necessary. Thinger.io has its own modeling language that facilitates the modeling of these services in a transparent way to the designer, being supported by the use of efficient communication protocols and a highly scalable architecture.

## 3. Thinger.io Overview

In this section, we are going to depict the general architecture of the new Open Source platform for deploying data fusion applications by integrating Big Data, Cloud and IoT Technologies. Thinger.io is a new platform that is receiving interest by the scientific/technological community, finding projects in which this platform has been used successfully [26,27,28,29,30,31], including in the field of education [32]. Thinger.io provides a ready to use cloud service for connecting devices to the Internet to perform any remote sensing or actuation over the Internet. It offers a free tier for connecting a limited number of devices, but it is also possible to install the software outside the cloud for a private management of the data and devices connected to the platform, without any limitation.

**Figure 1:**
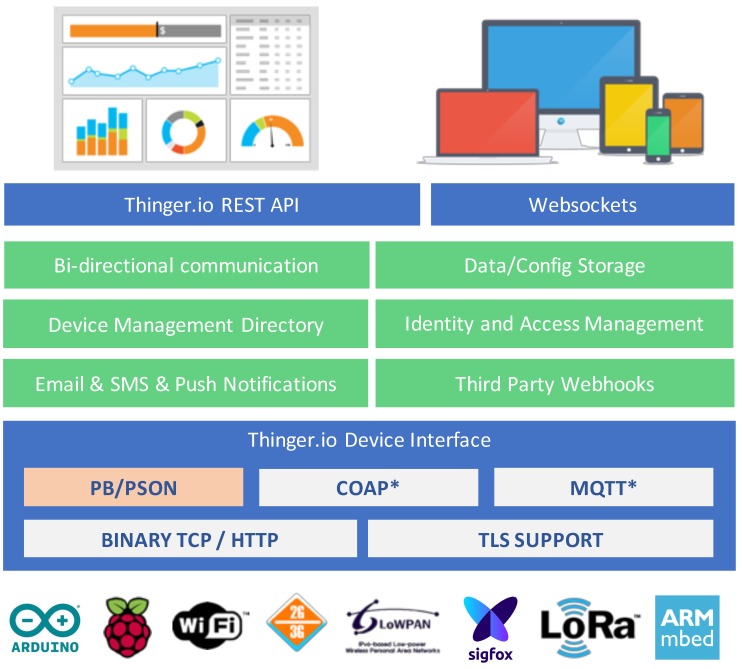
General overview of Thinger.io.

This platform is hardware agnostic, so it is possible to connect any device with Internet connectivity, from Arduino devices, Raspberry Pi, Sigfox devices, Lora solutions over gateways, or ARM devices, to mention a few. The platform provides some out-of-the-box features such as device registry; bi-directional communication in real-time, both for sensing or actuation; data and configuration storage, so it is possible to store time series data; identity and access management (IAM), to allow third party entities to access the platform and device resources over REST/Websocket APIs; third party Webhooks, so the devices can easily call other webservices, send emails, SMS, push data to other clouds, etc. It also provides a web interface to manage all the resources and generate dashboards for remote monitoring. The general overview of this platform is available in Figure 1.

The main benefit of using this platform, aside from the fact that it is open source, is the possibility to obtain a bi-directional communication with the devices, in real time, by using standard REST-API interfaces. As such, it is possible to develop any data fusion application, i.e., desktop, mobile, Webservice, that interacts with devices by using a well-known and proven interface based on REST. Meanwhile, the devices can use more efficient (in terms of bandwidth, or memory footprint) binary protocols to communicate with the cloud. Additionally, this platform provides client libraries for connecting several state-of-the-art IoT devices such as Arduino-based, ESP8266, ESP32, LinkitOne, Texas Instruments CC3200, Libellium Waspmotes, Raspberry Pi, etc. The client libraries provide a comprehensive way of connecting devices and sending information to the cloud, without having to deal with complex IoT protocols.

### 3.1. Research Efforts at Thinger.io

One of the key differences of the proposed solution, aside from being an open source platform, is the design of a specific solution and protocols for the IoT. These protocols have been designed thinking on IoT applications and use cases, covering the mentioned IoT challenges. In the following sections are disucssed the research efforts at Thinger.io and the main differences with other approaches.

#### 3.1.1. Transmission Efficiency

Most of the state-of-the-art IoT platforms use traditional transport protocols based on HTTP (Hypertext Transfer Protocol) or MQTT (Message Queuing Telemetry Transport) for pushing data from devices to the cloud [33,34]. In the HTTP approach, every device sending data to the cloud issues a HTTP request with a custom payload. The IoT platform receives this information, just like a regular web server, and stores this information in a database. This way of communication results in an inefficient way of transmitting information, in terms of bandwidth, latency, and power consumption, which are constraints for IoT devices [35]. As such, every sensed point requires the creation of a new connection to the cloud, and the creation of a HTTP request which includes several headers and a payload, usually in JSON or XML. This mechanism, based on traditional HTTP requests, implies a huge overhead for sending small and regular payloads, which in most cases, consists of a few bytes with sensor measurements. This is the preferred approach for other open and standard solutions, such as Fiware, a cloud-based infrastructure for IoT platforms funded by the European Union and the European Commission, as seen in multiple developments [36,37]. There are other solutions such as Xively [38], ThingSpeak [39], or Temboo [40], which all started offering only HTTP interfaces.

There are other IoT approaches based on protocols like MQTT [41], which is a telemetry protocol designed decades ago that provides a bidirectional communication between servers and devices, based on a publish-subscribe-based messaging protocol. This approach is much more efficient than HTTP-based solutions, as discussed in [35], as it scales better, requires less bandwidth, and reduces latency. Big companies such as Amazon Web Services are basing their IoT solutions on this protocol [33]. Nowadays, almost all IoT players started to integrate MQTT in their solutions, including the ones mentioned above, and others like Kaa, Carriots, or Ubidots [42].

Thinger.io proposes a similar efficient approach to the MQTT solution, in the sense of using raw binary connections without the HTTP overhead, or being able to provide publish-subscribe mechanisms. It also provides features not found on MQTT, like transparent HTTP interoperability, as described later, but in terms of transmission efficiency, it also defines the paylaod encoding (as opposed to MQTT). It uses an optimized encoding scheme called Protoson (PSON) (https://github.com/thinger-io/Protoson). Protoson was designed from scratch just to support devices with constrained resources such as memory or processing power.

**Figure 2:**
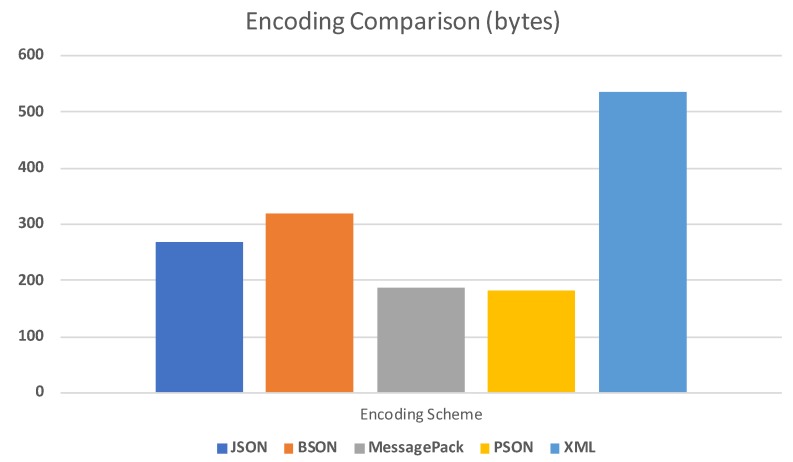
Encoding size comparison between different formats. Thinger.io uses a PSON encoding scheme to improve memory footprint, save bandwidth, and reduce power consumption in devices.

This solution allows encoding unstructured data like in JSON, but in a compact binary format. Figure 2 provides a comparison of encoding sizes between different common formats used by platforms, such as JSON, BSON (Binary JSON), MessagePack, XML, and the proposed PSON, which performs slightly better than the most efficient alternative (MessagePack), which is not quite common in the IoT domain. As such, the proposed solution reduces latency while sending information to the cloud, saves bandwidth, reduces memory footprint, and save battery by offering efficient protocols and an encoding mechanism.

#### 3.1.2. Real-Time Bidirectional Communication

HTTP-based platforms can be easy to develop, deploy, and to maintain, as they operate as regular HTTP servers receiving data from sensors using well-known technology. However, such approaches cannot provide an efficient mechanism for a bidirectional communication, i.e., when it is necessary to actuate over a device, or configure it in real time. HTTP-based platforms rely on a polling mechanism where the devices check the server periodically for new commands or updates. Meanwhile, Thinger.io provides a bidirectional communication channel between the device and the cloud server, so any application can interact with the device in real time, increasing the number of use cases that can be developed with the platform.

The other solutions based on MQTT also provide bidirectional communication between servers and devices. However, this protocol is only focused on the transport layer, allowing different endpoints to communicate, but does not define a language for modeling information like the proposed protocol in this paper; does not provide an efficient encoding schema like Protoson; or does not allow an easy interoperability with third party applications and services, i.e, over a REST API to query information in real time from the device.

Thinger.io addresses all these issues, as the underlying protocols support bidirectional communication, with an efficient encoding schema, and moreover, it also solves the interoperability of the devices with other applications, as described in the following subsection.

#### 3.1.3. Interoperability

One of the key benefits of Thinger.io is the possibility of being interoperable with other platforms and applications. That means that any device is accessible, both for sensing and actuating, from standard REST APIs, hiding the complexity of the underlying protocol optimizations between devices and the server.

**Figure 3:**
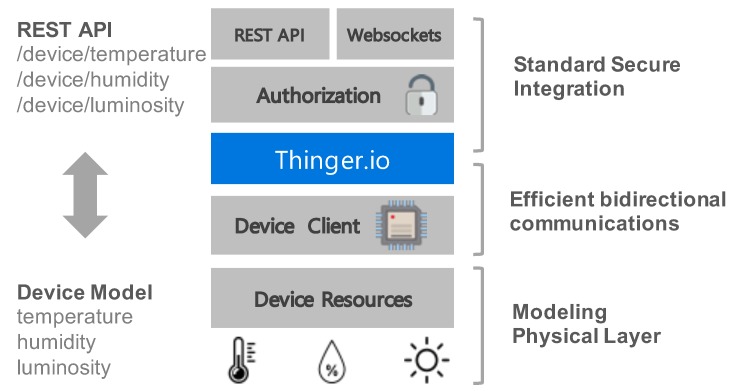
From the device model to REST API. Thinger.io allows device models to be defined that are automatically converted to REST APIs and can be consumed from any external application in real time.

The REST endpoints to talk with devices are not defined statically, or manually set in the server. Instead, they are dynamically extracted from the device model definition when it is connected to the cloud. So, a set of resources defined in the device code can be accessed in real time from the generated REST endpoints. Figure 3 illustrates this use case, where a device contains a set of sensors (temperature, humidity, luminosity) that are defined in the device model. Using the Thinger.io client libraries, the device can connect to the Thinger.io cloud, using efficient bidirectional communications. Once the device is connected, the resources defined in the model can be accessed in real time from the automatically generated REST endpoints, like */device/temperature*, that executes the corresponding resource to obtain the current sensor reading.

There are other use cases where applications require periodical updates, i.e., sampling accelerometers in real time, or giving temperature updates every second. Polling this kind of data over REST APIs is not efficient, as it generates too much overhead in the communication, and adds extra latency as it is necessary to issue a request every time a resource update is necessary. Thinger.io also provides mechanisms for subscribing to resource updates over standard Websockets. Thus, an external client can open a Websocket to the platform, and then, subscribe to a resource at a requested interval.

This approach differs from other IoT solutions, especially from HTTP-based solutions where devices just push data to the cloud, and an external client consumes this information. In such a case, there is no real-time interaction between the external client and the device, as it is limited by the push or poll synchronization periods configured in the device. It also differs from MQTT platforms, as MQTT does not allow data models to be defined that can be exposed directly to standard REST APIs.

#### 3.1.4. Modeling Simplicity

Finally, one of the key aspects of this platform, and the main focus of this paper, relates to the ability of modeling information in devices in an easy way. There are several IoT solutions out there that require an extensive coding to do simple tasks like sending a sensor reading, or parsing an input command on the device. This coding usually involves managing HTTP requests, adding headers, parsing input data, etc., so a simple task usually requires dozens of lines of code.

In this case, Thinger.io provides client libraries that simplify modeling any kind of device resources, like input resources for actuation over the device; output resources to send information generated in the device; resources that require an input and generate an output; or execution functions that can be triggered remotely. As such, it is possible to cover multiple use cases, in a simple way, requiring just a small coding.

**Figure 4:**
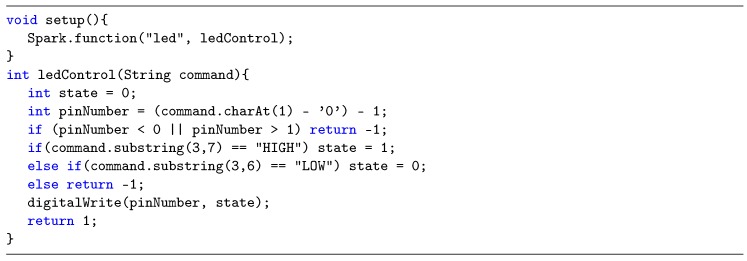
Particle.io code example for enabling/disabling an arbitrary digital pin.

Just to provide a comparative example, Figure 4 presents a sample code from the Particle platform, which is a state-of-the-art IoT vendor, known for its simplicity. In this example, the *Setup* code registers a function in the cloud, called *led*, that allows an led to be turned on and off remotely. The body of this function, called *ledControl*, just receives a *String* command, that needs to be parsed to extract both the pin number and the desired state. So, a simple requirement like turning on/off a digital pin becomes complex to code, leading to the possibility of undesired bugs being included.

**Figure 5:**
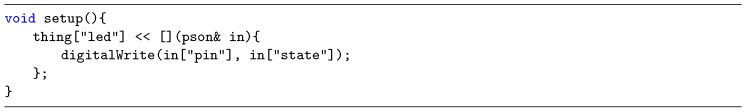
Thinger.io code example for controlling an arbitrary digital pin.

Additionally, Thinger.io provides a modeling language based on Protoson, which is just like JSON but much more efficient. Modelling the same functionality that this one described for the Particle platform requires just a couple of simple lines. This example is provided in Figure 5, where a resource is defined called *led*, which exposes an input function that just does a *digitalWrite* over the parameters given in *pin* and *state*.

**Figure 6:**
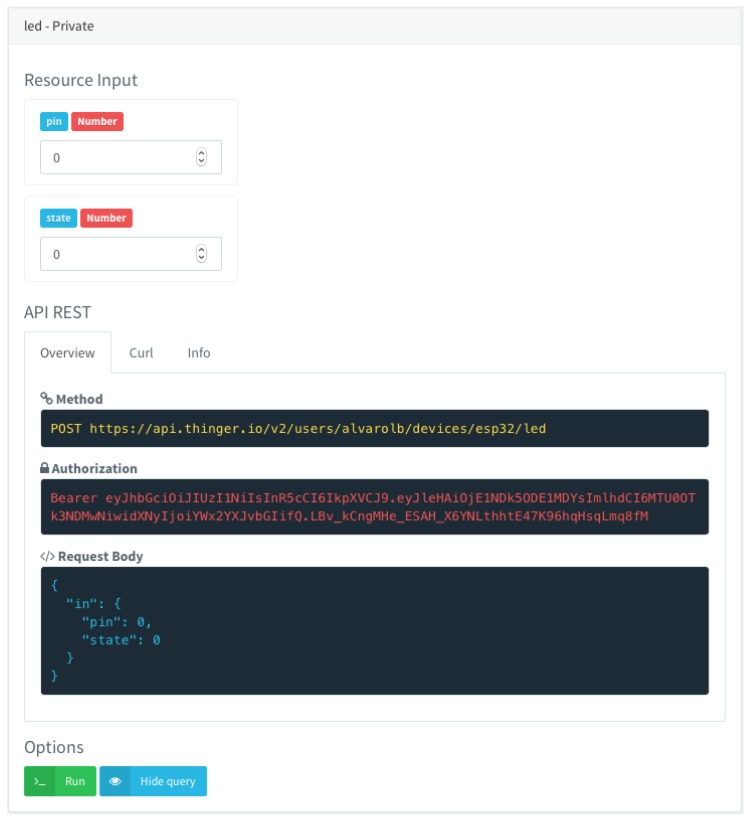
Example of how a resource definition modelled in the device, like turning on/off a digital pin, is automatically converted to an REST API endpoint that can be executed directly from the console or any other third party application.

Moreover, such simple definition in the device code, as described in the interoperability subsection, automatically generates a REST API endpoint that can be used from the Cloud, including also the expected fields to execute the command, which are extracted automatically from the model definition. This endpoint can be easily tested from the console client provided by Thinger.io, as shown in Figure 6.

### 3.2. Storage Management

**Figure 7:**
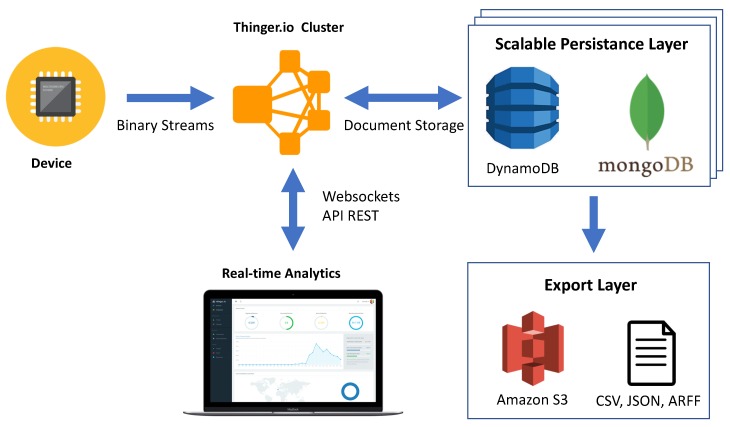
Thinger.io Big Data Storage Overview.

One of the features in this platform is related with the storage management, which is called “Data Buckets”. A bucket is a cloud resource for storing time series data. A data bucket is a time series storage where devices can push information when required. Each data point is automatically timestamped in the cloud at reception time, as IoT devices do not handle a real-time clock (RTC) by themselves. This information is stored in the cloud in secure, efficient, and scalable solutions (mainly DynamoDB from Amazon Web Services). The information stored in a bucket can be both displayed on a dashboard inside the console interface or exported in scalable storage (like Amazon S3) in ARFF, JSON, or CSV for its offline analysis, as illustrated in Figure 7.

The storage of information in a scalable cloud infrastructure like Thinger.io can be divided in the following steps:

1. Model data resources on the device. A device can expose a set of resources, i.e., temperature or humidity. The resources are not tied to any data structure, as they are managed as non-structured documents, like in JSON format. Modelling such resources is covered in Section 4.
2. Connect the device to Thinger.io infrastructure. The device exposes the available resources, that are immediately available both for feeding real-time dashboards, or storing them in data buckets.
3. Configure a Bucket in Console. Once the device is connected to the platform, it is possible to define a new data bucket that is fed by a device resource. The user normally selects here the sampling interval required. In this moment, the device will start to stream the information at the required frequency.

In this approach, the Thinger.io cloud infrastructure automatically subscribes to the device resources at the required interval, as long as there are sinks for this information (a dashboard, or a data bucket). In the case of storing the information in data buckets, the platform supports the use of different document storages, like DynamoDB or MongoDB. Both alternatives provide highly scalable solutions for storing data in the Cloud, as they natively support sharing. Moreover, the Thinger.io infrastructure automatically generates OLAP (On-Line Analytical Processing) cubes for providing metrics like average, sum, count, max, and min, aggregated by different time periods. All of this information can be used then for the mentioned analytics dashboards, or for exporting for data analysis. Thinger.io relies on scalable cloud storage for this part, as it supports export to Amazon S3, which can contain files up to 5 TB.

## 4. Modelling Data Resources with Thinger.io Platform

The aim of this section is to show the capabilities of Thinger.io to model device data resources, which are necessary to integrate the Cloud, Big Data and IoT technologies. Thinger.io provides both the technological environment for sensors connectivity, as well as certain hardware devices that allow the task of integration and connectivity of the sensors to be simplified using development environments known as Arduino.

### 4.1. Sketch Overview

The sketch is formed by two sections: a setup method and a loop method. In general, any device resource (led, relay, sensor, servo, etc.) must be defined inside the setup() method. It is the section where the variables are also initialized, an input/output direction of a digital pin is set on, or the Serial port speed is initialized. This basically consists on configuring what values or resources we want to expose over the Internet. On the other hand, the loop() method is the place to always apply the thing.handle() method, so the thinger libraries can handle the connection with the platform. This is the place also to apply other platform resources such as ndpoints, or stream real-time data to an open WebSocket.

**Figure 8:**
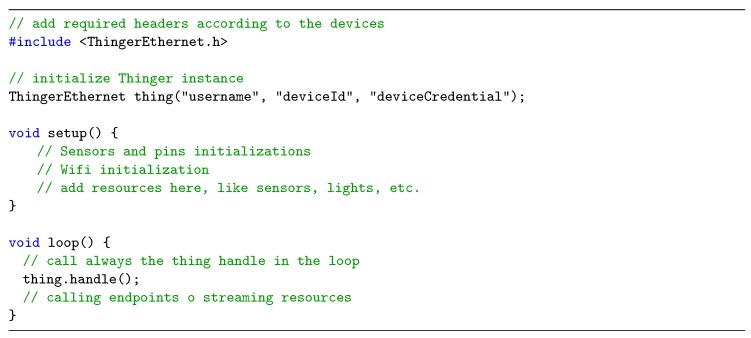
The basic sketch of a Thinger.io program.

All of the devices connected to the platform need to be authenticated against the server. First, we need to create a device in the console; we are basically creating a new device identifier and setting a device credential. In the next section, we will see a proof of concept showing how these credentials are obtained. Therefore, It is necessary to also set up these credentials in the code, so the device can be recognized and associated to our account. This is normally done while initializing the Thinger instance in the code. Figure 8 depicts the basic sketch of a Thinger.io program.

### 4.2. Defining Resources

In the Thinger.io platform, each device can define several resources. We can consider a resource to be anything we can sense or actuate. For example, a typical resource will be a sensor value like temperature or humidity, or a relay that turns on and off a light. We should define the resources we need to expose over the Internet. All resources must be defined inside the setup() method. The resources are configured at the beginning, but can be accessed later as necessary. There are three different types of resources, which are explained in the following sections.

#### 4.2.1. Input Resources

To define an input resource, the operator *<<* pointing to the resource name is used, and it uses a C++11 Lambda function to define the function. The input resource function takes one parameter of type pson that is a variable type that can contain booleans, numbers, floats, strings, or even structured information like in a JSON document. The following subsections will show how to define different input resources for typical use cases.

#### Turn on/off a Led, a Relay, etc.

**Figure 9:**
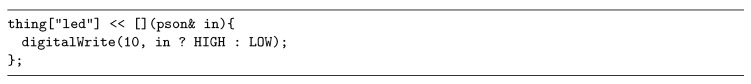
Turn on/off the digital pin 10.

This kind of resource only requires an on/off state so it can be enabled or disabled as required. As the pson type can hold multiple data types, we can consider the pson parameter of the input function as like a boolean. So, inside the setup function, we can place a resource called led (or other name), of input type (using the operator <<), that takes a reference to a pson parameter. Figure 9 depicts an example code that will turn on/off the digital pin 10 using a ternary operator over the in parameter.

#### Modify a Servo Position

**Figure 10:**
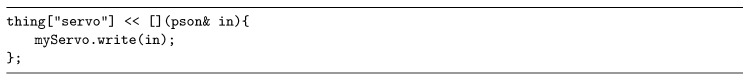
Modify a servo position.

Modifying a servo position is quite similar to turning on/off a led. In this case, however, it is necessary to use an integer value. As the pson type can hold multiple data types, we can still use the pson type as an integer value. Figure 10 depicts an example of modifying a servo position.

#### Update Sketch Variables

**Figure 11:**
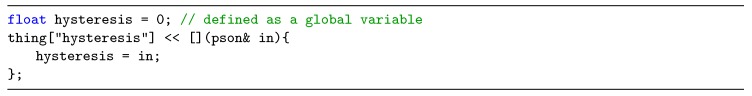
Update sketch variables.

We can also use the input resources for updating our sketch variables, so we can change our device behaviour dynamically. This is quite useful in some situations where we want to temporarily disable an alarm, change the reporting intervals, update a hysteresis value, and so on. In this way, we can define additional resources to change our variables (see Figure 11).

#### Pass Multiple Data

**Figure 12:**
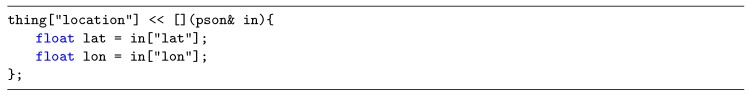
Pass multiple data.

The pson data type can hold not only different data types, but is also fully compatible with JSON documents. So, we can use the pson data type to receive multiple values at the same time. Figure 12 depicts an example which will receive two different floats that are stored with the lat and lon keys.

#### 4.2.2. Output Resources

Output resources should be used in general when we need to sense or read a sensor value, such as temperature, humidity, etc. So the output resources are quite useful for extracting information from the device. To define an output resource, the operator >> is used, pointing out the resource name, and it uses a C++11 Lambda function to define the output function. The following subsections will show how to define different output resources for typical use cases.

#### Read a Sensor Value

**Figure 13:**
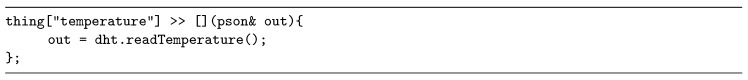
Read a sensor value.

Defining an output resource is quite similar to defining an input resource, but in this case the operator >> is used. In the callback function, we can fill the output value with any value we want—in this case, the output from a sensor reading (see Figure 13).

#### Read Multiple Data

**Figure 14:**
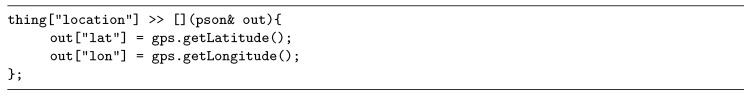
Read multiple data.

In the same way that the input resources can receive multiple values at the same time, the output resources can also provide multiple data. This is an example for providing both latitude and longitude from a GPS (see Figure 14).

#### 4.2.3. Input/Output Resources

**Figure 15:**
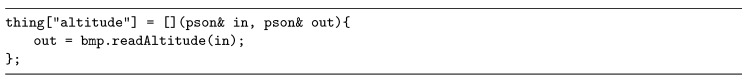
Input/Output Resources.

The last resource type is a resource that not only takes an input or an output, but takes both parameters. This is quite useful when we want to read an output that depends on an input, i.e., when we need to provide a changing reference value to a sensor. This kind of resource is defined with the operator =. In this case, the function takes two different pson parameters: one for input data and another for output data. Figure 15 depicts an example which provides an altitude reading using the BMP180 Sensor. It takes the reference altitude as input, and provides the current altitude as output.

At this point, we have shown some of the basic characteristics of the Thinger.io language that facilitate the modeling of applications in the field of IoT. In http://docs.thinger.io/arduino/#coding, we can find more advanced resources such as special macros, communication between devices, use of endpoints, use of streaming data, etc.

## 5. A Case of Use of Meteorological Information Service Modeling

In this section, the modeling simplicity of a meteorological service will be checked. For the development of this service, it is necessary to manage data coming from the weather station (Big Data), and connectivity with the meteorological station (IoT), with the storage and management of the information collected in a Cloud environment.

### 5.1. Hardware

**Figure 16:**
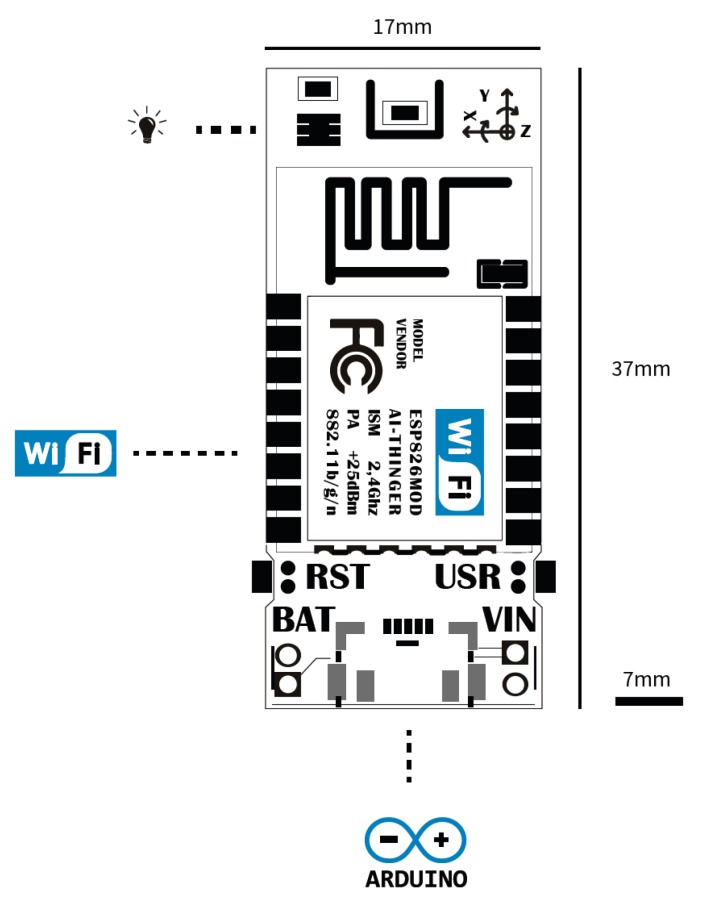
Thinger.io logical interface.

**Figure 17:**
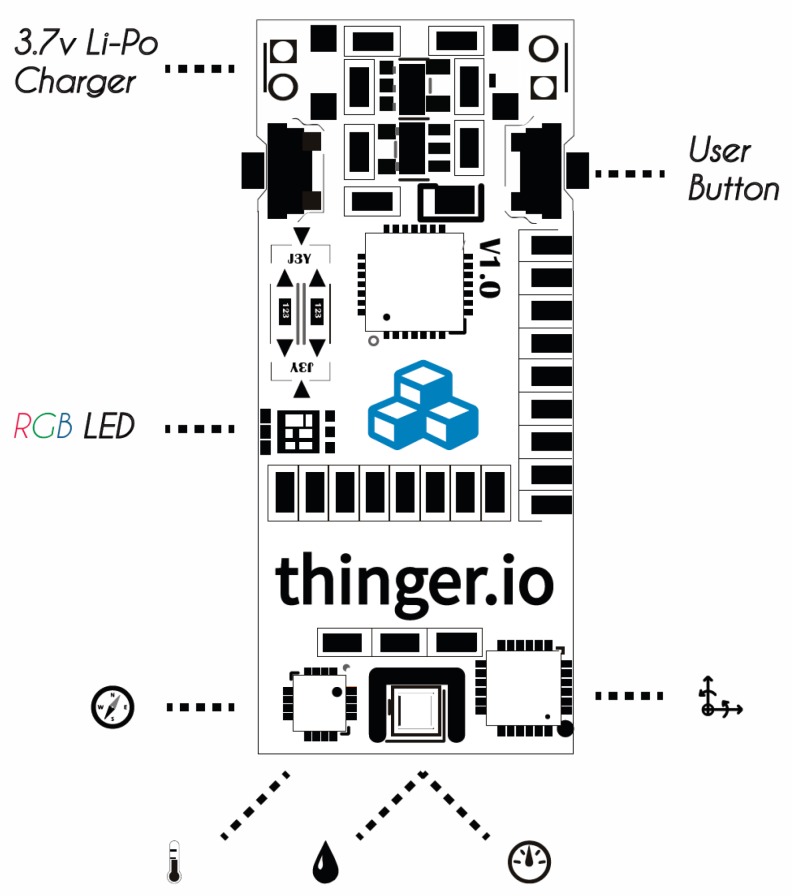
Thinger.io physical interface.

For the development of this case study, a hardware device of the Thinger.io platform will be used, called ClimaStick, which integrates both the sensors and Internet connectivity. This device will allow us to collect weather information over time, without having to have knowledge of electronics, data buses, sensors, etc. The device is based on an Espressif ESP8266 microcontroller, which has a Cortex M0 at 80 Mhz, 4 MB of flash, and 160 KB of RAM. This board also has 802.11 b/g/n WiFi connectivity for its Internet connection, as well as serial port interfaces through USB that allows its programming through the Arduino development environment. The development plate has small dimensions of 37 × 17 × 5 mm (Figure 16 and Figure 17).

**Figure 18:**
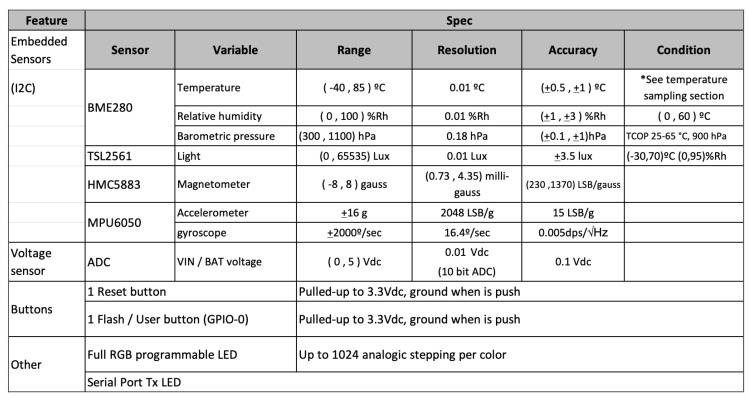
Integrated sensors in Thinger.io.

This board integrates sensors that allow measuring the temperature, relative humidity, barometric pressure, or brightness, necessary for the development of the practical case. It also integrates other sensors, such as a magnetometer, an accelerometer, and a gyroscope, which can be useful for other practical cases where it is necessary to have inertial sensors. The sensors used by this device, as well as its operating ranges, resolution, and accuracy, are specified in Figure 18.

### 5.2. Platform Configuration

In this section, it will be shown how we can model and configure a device within the Thinger.io platform. After registering on the platform Thinger.io, it will be necessary to register two resources within the administration panel. In this case, we will have to register a device, which will be the one that connects to the platform to provide the information of the sensors (in our case, the ClimaStick), and we will also have to register a data bucket that allows the information to be saved over time. The management of these resources is documented in more detail at http://docs.Thinger.io/console/, but here the basic steps necessary for the development of the case study are provided. To create the device, we will access the *Devices* section of the side menu of the platform, from which we can manage the devices associated with our user account.

**Figure 19:**
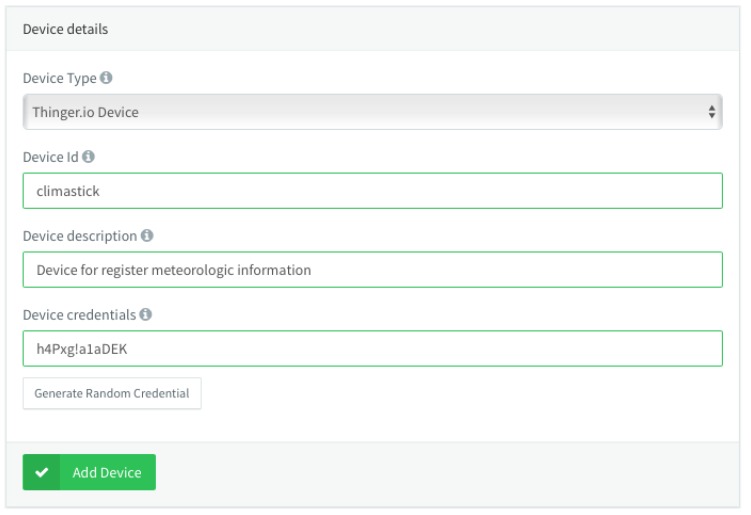
Adding a device in Thinger.io.

Once we are in this section, it will be necessary to register the device by clicking the *Add Device* button in the list of devices, which will open a section to record basic information about the device, such as its identifier, a description, and its access credentials. It is important to keep the information of the identifier and the access credentials, because then it will be necessary to use it in the code that will be programmed in the device. For this practical example, the identifier of the device is *Climastick*, and its credentials are h4Pxg!a1aDEK, as can be seen in Figure 19.

To create the data bucket, analogously, we must go to the corresponding section of the administration console, called *Data Buckets* in this case.

**Figure 20:**
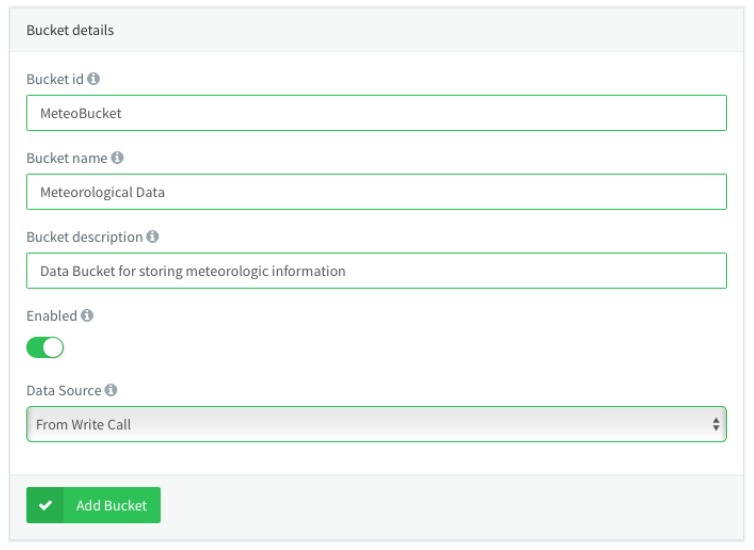
Data bucket configuration in Thinger.io.

Once we are in this section, it will be necessary to register the data bucket by clicking the *Add Bucket* button in the data bucket list, which will open a section to configure the information store. In the same way as with the device, it will be necessary to assign an identifier of the data bucket, i.e., a name and a description, if we want to activate it, or the source of data from which the information will be taken. In this case, we will create a data bucket with the identifier *MeteoBucket*, which will be activated, and the data source will be *From Write Call*, that is, the device will start writing the information when it deems necessary. Figure 20 depicts an example of the mentioned data bucket configuration.

With these two resources created, we can now prepare our device so that, on the one hand, it connects to the platform using the generated credentials, and on the other, it sends weather information to the data bucket.

### 5.3. Modeling Devices

In this section, we will show how to model the behavior of physical devices so that they send the information to their corresponding data bucket. In this way, once the platform has been configured to support the connection of a new device, and a data bucket has been created to store the data, it is necessary to program the device to connect to the platform, read the information of the sensors, and write in the data bucket. The programming of the device will be done through the Arduino environment, for which it is necessary to carry out some adjustments to use the ClimaStick libraries, and allow the programming of the ESP8266 microcontroller on which it is based. This initial process is documented in detail within the platform documentation at http://docs.Thinger.io/hardware/climaStick/ (in the environment configuration section), and it is similar to the process of adding any other board within the Arduino environment.

**Figure 21:**
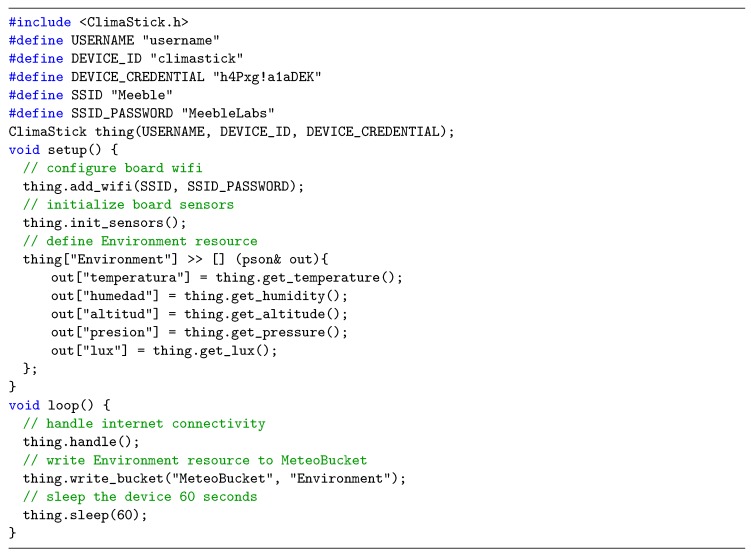
An example of modelig a device in Thinger.io.

We only need to model the Climastick device in order to send the information to the data bucket that we have defined in the Thinger.io platform. The way to model is through the simple programming language incorporated in Thinger.io and it has been described in the previous section (Figure 21).

In this code example, the following parameters are being defined:

- **USERNAME** It is the username that we have chosen in the process of registering our account.
- **DEVICE_ID** It is the identifier of the device that we use in the registration process, in our example ‘climastick’.
- **DEVICE_CREDENTIAL** It is the credential of the device that we use in the process of registering the device, in our example h4Pxg!a1aDEK.
- **SSID** It is the name of the WiFi network to which the device will be connected; the one used in this example would be ‘Meeble’.
- **SSID_PASSWORD** It is the password of the WiFi network to which the device will be connected, which would be ’MeebleLabs’ in the example.

With the USERNAME, DEVICE_ID, and DEVICE_CREDENTIAL parameters, a variable called thing of type ClimaStick is being initialized, which represents our device, on which we can configure the WiFi connection, as well as read the information from the sensors. Subsequently, within the Setup method (which is executed only once at the beginning of the program), it is being done:

- Initializing the WiFi connection of the device with the SSID parameters, and SSID_PASSWORD through the thing.add_wifi method.
- Initializing the sensors of the device through the call init_sensors ().
- Defining a resource within the device called Environment, which will contain the meteorological information that we want to store, which in this case is temperature, humidity, altitude, pressure, and luminosity.

Finally, within the loop method, which is executed indefinitely, after the setup, the following is being done:

- The management of the connection of the device to the platform through the call thing.handle (), where it checks whether the device is connected to the Internet, authenticates the device, and keeps it connected when necessary.
- The writing of the Environment resource defined in the setup, to the MeteoBucket data store previously registered in the platform. For this, the thing.write_bucket () method is used.
- Pause the device for a certain period of time (in this case, 1 minute, but can be adjusted as necessary) through the thing.sleep () method. This method does not delay the execution of the next iteration; it basically sleeps the device, the sensors, and the wireless connection radio. The device restarts after the specified time. In this way, the device can be powered through a battery.

**Figure 22:**
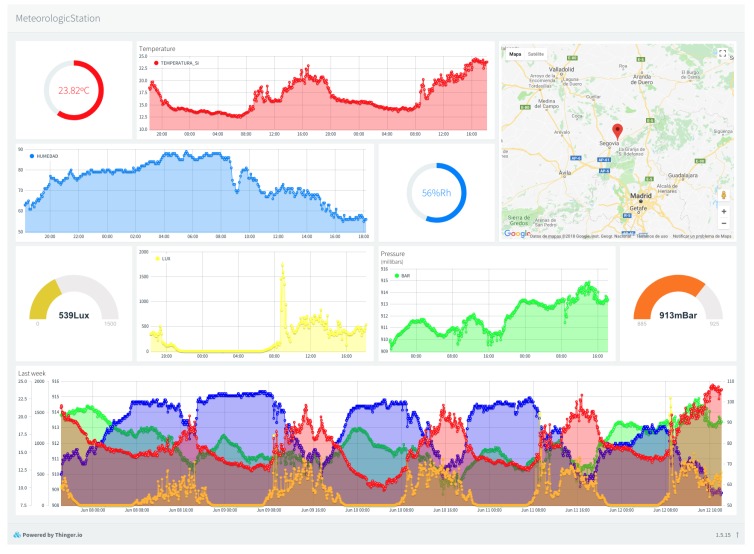
An example of the console of Thinger.io.

In Figure 22, a screenshot of the console of Thinger.io is shown with the values that have been stored by each of the declared resources.

## 6. Conclusions

In this paper, the authors presented a new open source platform for modeling data fusion applications based on IoT, Big Data and cloud technologies. This new ecosystem will facilitate the deployment of new data fusion applications in different fields, including smart homes and buildings, vehicular systems and transportation, medical and health care systems, smart infrastructures, power grids, and Industry 4.0, among others. While developing this platform, we had three different goals. The first one was to provide an open source platform that can be maintained, evaluated and tested by the community. Why rely on a single team when we can rely on a worldwide team? There are many benefits to this approach, but the most noteworthy is the confidence—the confidence on tested software; the confidence on inspected code; the confidence that we can take the source code and adapt it to our needs; the confidence that multiple people, instead of a single vendor, maintain the software. That is the goal of making the Thinger.io platform open source. The second goal was to make the platform hardware agnostic. A good platform must support as many devices as possible—from devices with very low computing capabilities to whole computers with an operating system; from devices using a wired connection to devices using any wireless technology. The user or the expert designing the IoT product makes the final choice. Finally, one of our main goals concerned the people behind the modeling of the IoT ecosystem. We want to provide a truly special modeller experience, so they can spend their time designing and providing special user experiences. Everybody appreciates a really good design. In the paper, emphasis is given to the modeling capabilities of Thinger.io, which allows a simple deployment of services that integrate Cloud, Big Data and IoT technologies.

In future lines of work, the authors are working to develop a graphical interface that allows the graphical modeling of data fusion applications where the integration of IoT, Cloud and Big Data technologies is necessary. This ability will allow the use of the Thinger.io tool for people without excessive programming knowledge, making its use more widespread.
